# Relationship between fibroblastic foci profusion and high resolution CT morphology in fibrotic lung disease

**DOI:** 10.1186/s12916-015-0479-0

**Published:** 2015-09-24

**Authors:** Simon L F Walsh, Athol U. Wells, Nicola Sverzellati, Anand Devaraj, Jan von der Thüsen, Samuel A. Yousem, Thomas V. Colby, Andrew G. Nicholson, David M. Hansell

**Affiliations:** Department of Radiology, Royal Brompton Hospital, Sydney Street, London, SW3 6NP UK; Interstitial Lung Diseases Unit, Royal Brompton Hospital, Sydney Street, London, SW3 6NP UK; Department of Clinical Sciences, Section of Radiology, University of Parma, Via Gramsci 14, 43126 Parma, Italy; Department of Radiology, St Georges Hospital, Tooting, London, SW17 0QT UK; Medisch Centrum Haaglanden, The Hague Area, Netherlands; Department of Pathology, University of Pittsburgh, Pittsburgh, Pennsylvania USA; Department of Laboratory Medicine and Pathology, Mayo Clinic, Scottsdale, Arizona USA; Department of Histopathology, Royal Brompton Hospital, Sydney Street, London, SW3 6NP UK; Department of Radiology, Kings College Hospital Foundation Trust, Denmark Hill, London, SE5 9RS UK

**Keywords:** Interstitial lung disease, Idiopathic pulmonary fibrosis, Fibroblastic foci, Bronchiectasis, Survival

## Abstract

**Background:**

Fibroblastic foci profusion on histopathology and severity of traction bronchiectasis on highresolution computed tomography (HRCT) have been shown to be predictors of mortality in patients with idiopathic pulmonary fibrosis (IPF). The aim of this study was to investigate the relationship between fibroblastic foci (FF) profusion and HRCT patterns in patients with a histopathologic diagnosis of usual interstitial pneumonia (UIP), fibrotic non-specific interstitial pneumonia (NSIP) and chronic hypersensitivity pneumonitis (CHP).

**Methods:**

The HRCT scans of 162 patients with a histopathologic diagnosis of UIP or fibrotic NSIP (n = 162) were scored on extent of groundglass opacification, reticulation, honeycombing, emphysema and severity of traction bronchiectasis. For each patient, a fibroblastic foci profusion score based on histopathologic appearances was assigned. Relationships between extent of fibroblastic foci and individual HRCT patterns were investigated using univariate correlation analysis and multivariate linear regression.

**Results:**

Increasing extent of reticulation (*P* < 0.0001) and increasing severity of traction bronchiectasis (*P* < 0.0001) were independently associated with increasing FF score within the entire cohort. Within individual multidisciplinary team diagnosis subgroups, the only significant independent association with FF score was severity of traction bronchiectasis in patients with idiopathic pulmonary fibrosis (IPF)/UIP (n = 66, r^2^ = 0.19, *P* < 0.0001) and patients with chronic hypersensitivity pneumonitis (CHP) (n = 49, r^2^ = 0.45, *P* < 0.0001). Furthermore, FF score had the strongest association with severity of traction bronchiectasis in patients with IPF (r^2^ = 0.34, *P* < 0.0001) and CHP (r^2^ = 0.35, *P* < 0.0001). There was no correlation between FF score and severity of traction bronchiectasis in patients with fibrotic NSIP. Global disease extent had the strongest association with severity of traction bronchiectasis in patients with fibrotic NSIP (r^2^ = 0.58, *P* < 0.0001).

**Conclusion:**

In patients with fibrotic lung disease, profusion of fibroblastic foci is strikingly related to the severity of traction bronchiectasis, particularly in IPF and CHP. This may explain the growing evidence that traction bronchiectasis is a predictor of mortality in several fibrotic lung diseases.

## Background

Accurate prognostic evaluation in fibrotic lung disease is important because it guides management decisions. Over the past 15 years a relatively large body of literature has emerged reporting clinical, radiologic and histopathologic features and, more recently, biomarkers which may influence survival in several different fibrotic lung diseases [1–13]. A number of these studies have highlighted the importance of the fibroblastic focus as a manifestation of active lung injury and in the particular setting of idiopathic pulmonary fibrosis (IPF), fibroblastic foci profusion may predict physiologic decline and mortality [7, 8, 14]. Although a defining feature of IPF, fibroblastic foci are also present in fibrotic non-specific interstitial pneumonia (NSIP) and chronic hypersensitivity pneumonitis (CHP), albeit less profuse [15–17]. Most patients with fibrotic lung disease, however, do not undergo surgical lung biopsy, limiting the clinical utility of fibroblastic foci evaluation for prognostication purposes. In contrast, high resolution computed tomography (HRCT) plays an integral part in the evaluation of patients with diffuse lung diseases, and several studies have reported on the prognostic significance of several HRCT patterns including honeycombing [1, 2, 18–20] and, more recently, traction bronchiectasis [4–6, 18, 21, 22]. To date no attempts have been made to determine whether a relationship exists between fibroblastic foci profusion and HRCT patterns; therefore, the aim of this study is to determine if fibroblastic foci profusion is linked to any individual HRCT pattern. Patients with histopathologic confirmation of UIP, fibrotic NSIP and CHP were included in the study in order to capture a full range of fibroblastic foci profusion.

## Methods

### Study population

The study population was selected from a histopathologic database containing patients who had undergone surgical lung biopsy between 1979 and 2010 at the Royal Brompton and Harefield NHS Foundation Trust. Inclusion criteria for entry to the study were patients who 1) had undergone diagnostic surgical lung biopsy and had a histopathologic diagnosis of UIP, fibrotic NSIP or CHP and 2) had a multidetector HRCT examination performed within 3 months of the biopsy. Surgical lung biopsies and HRCTs were clinically indicated in all cases, and for the purposes of retrospective examination of these data, the local research ethics committee waived the need for review by an external NHS Research Ethics Committee. Furthermore, informed patient consent was not required by the local research ethics committee. Permission to access patient clinical information for the purposes of this retrospective evaluation of clinically indicated data was granted by the local ethics committee. For each patient, the diagnosis was determined based upon clinical, radiologic and histopathologic data according to current American Thoracic Society (ATS)/European Respiratory Society (ERS) guidelines [23]. Pulmonary function tests (PFTs) were recorded for each patient if they had been performed within 3 months of the biopsy date. The composite physiologic index (CPI) [18] was calculated in each of the patients for whom PFTs were available, according to the formula CPI = 91.0 - (0.65 × DLco) - (0.53 × percent predicted FVC) + (0.34 × percent predicted FEV1). This index captures the severity of physiologic impairment due to interstitial lung disease while excluding the contribution from emphysema [24].

### Histopathologic evaluation and fibroblastic foci scores

**Fig. 1:**
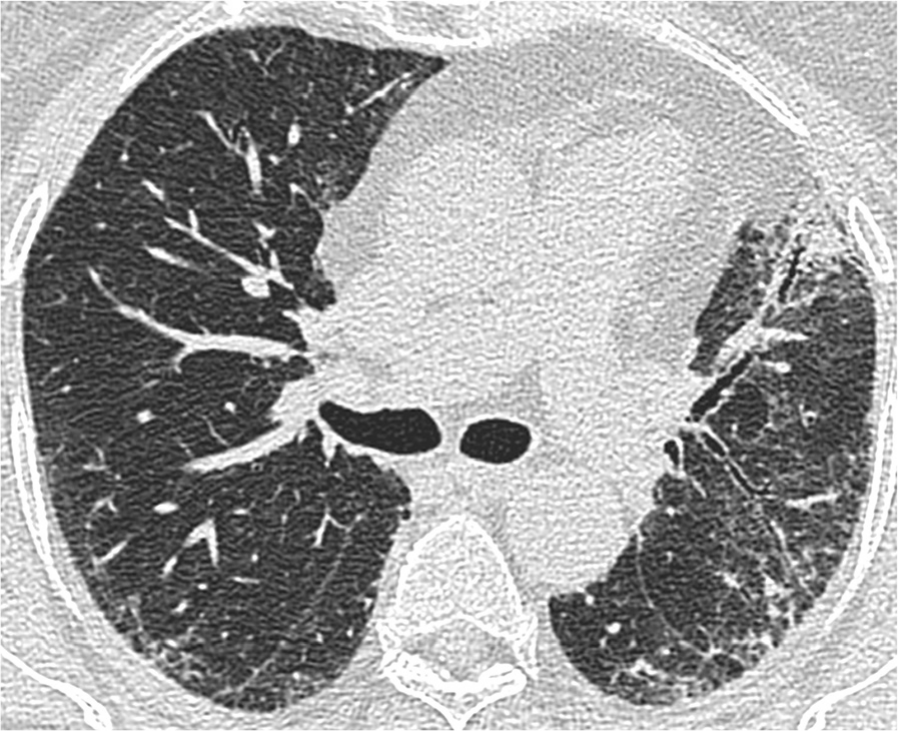
Biopsy proven fibrotic NSIP. MDT diagnosis: idiopathic fibrotic NSIP. Total disease extent at this level: 40 %. Ground glass opacification: 30 %, extent of reticulation 10 %, extent of consolidation 10 %. Traction bronchiectasis score in ground glass opacification: 0. Traction bronchiectasis score in consolidation: 1. Consensed FF score: 0.5

A semiquantitative evaluation of fibroblastic foci profusion (FF score) was performed using a scale of 0–6. All cases were scored by one pathologist (AN), and the second score was undertaken by an experienced pulmonary pathologist in relation to previous and current cohort studies (TC, SY, JvdT) [8]. A consensus diagnosis of histologic pattern was made for each case. Semiquantitative evaluation of fibroblastic foci profusion has been shown to correlate well with objective fibroblastic foci counts [8]. Absence of fibroblastic foci was scored as 0, and the most profuse score was 6 after the method described by Nicholson et al. ([8], see Fig. 1). In cases with biopsies from two different sites, an average score was taken. The average of each pathologist’s FF score was calculated to give an overall FF score for each patient.

### HRCT protocol and image evaluation

**Fig. 2:**
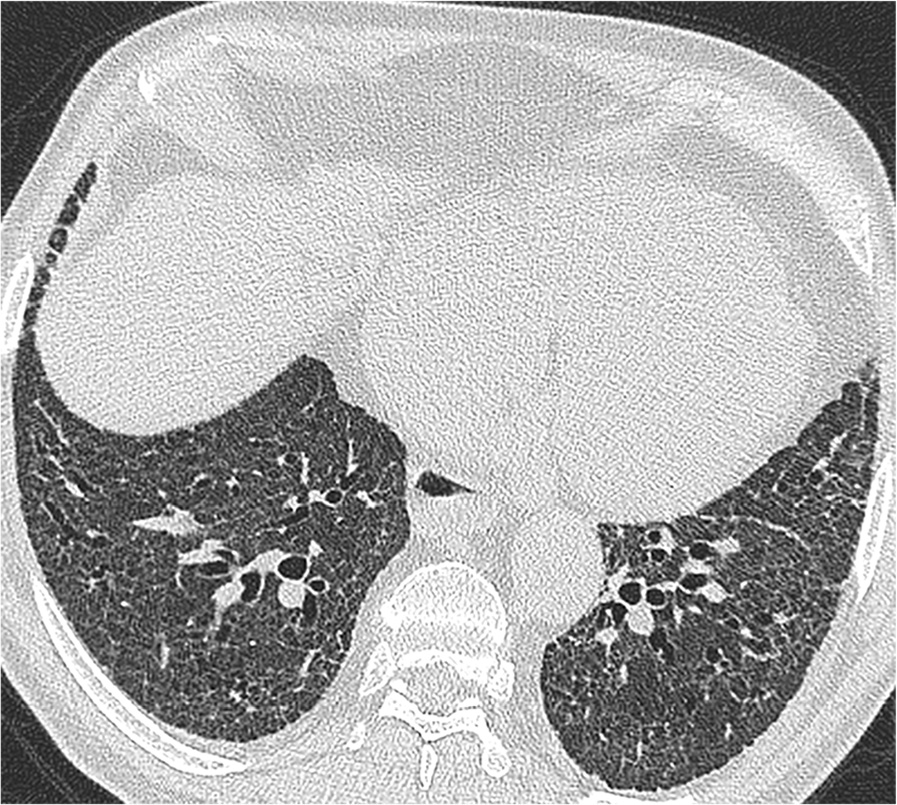
Biopsy proven fibrotic NSIP. MDT diagnosis: connective tissue disease related fibrotic NSIP. Total disease extent at this level: 95 %. Ground glass opacification: 90 %, extent of reticulation 10 %. Traction bronchiectasis scores in both patterns: 2. Consensed FF score: 0.5

**Fig. 3:**
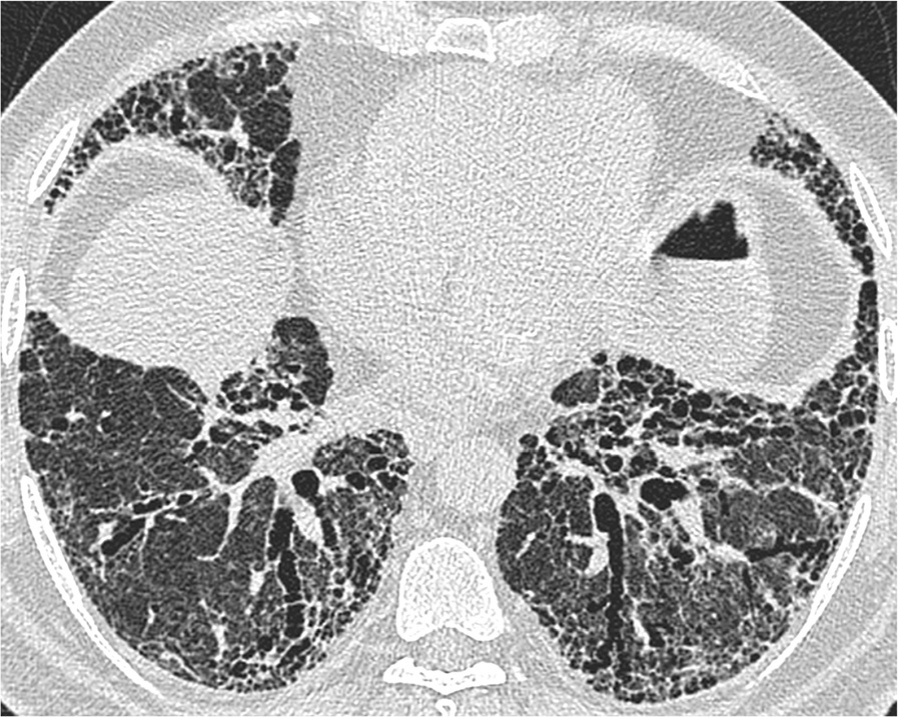
Biopsy proven UIP. MDT diagnosis: rheumatoid arthritis related UIP. Total disease extent at this level: 90 %. Ground glass opacification: 60 %, extent of reticulation 25 %, extent of honeycombing 15 %. Traction bronchiectasis scores in both patterns: 3. Consensed FF score: 3.5

A detailed description of the HRCT protocol, HRCT pattern definitions and HRCT scoring method can be found in the online repository. Two thoracic radiologists of 9 and 10 years’ experience scored HRCTs for each patient on the total disease extent, the extent of four interstitial patterns (ground glass opacification, reticulation, honeycombing and consolidation) and emphysema, at six levels. A score of the severity of traction bronchiectasis (0–3) was also assigned. At the end of scoring, each patient had a total disease extent score, for total extent scores for each of the four interstitial patterns, a total emphysema extent score and a total traction bronchiectasis score (examples are shown in Figs. 1, 2 and 3).

### Statistical analysis

Data are given as means with standard deviations (SD), medians with interquartile range (IQR) or as number of patients and percentage where appropriate. Statistical analyses were performed using STATA software (version 12, StataCorp, College Station, TX, USA). The single determination standard deviation was used to evaluate interobserver agreement for continuous variables (total interstitial disease extent, groundglass opacification, fine and coarse reticulation, honeycombing, consolidation and emphysema) [25]. The weighted kappa statistic (κ_w_) was used to evaluate interobserver agreement for categorical variables (traction bronchiectasis and FF scores) and was categorized as follows: poor (0 < κ_w_ ≤ 0.20), fair (0.20 < κ_w_ ≤ 0.40), moderate (0.40 < κ_w_ ≤ 0.60), good (0.60 < κ_w_ ≤ 0.80) and excellent (0.80 < κ_w_ ≤ 1.00) [25]. Univariate correlations between FF scores and HRCT variables were tested using the Spearman rank correlation analysis. Multivariate linear regression models were constructed to identify independent determinants of FF score using a backward elimination procedure, starting with all candidate variables, and at each step removing variables using a *P*value cut-off of 0.05 until no variables met this cut-off point criterion for removal. The assumptions of linear regression were tested and confirmed by inspection of residual-versus-predictor plots, and heteroskedasticity was tested for graphically (by inspection of residuals plotted against fitted values) and non-graphically (using the Cook-Weisberg test for heteroskedasticity). To investigate the impact of regression outliers, each multivariate equation was re-examined after the exclusion of observations generating the greatest equation leverage (identified by inspecting leverage-versus-residual squared plots).

## Results

### Patient population baseline characteristics

The initial population comprised 338 patients with semiquantitative scores for FF. Exclusions were due to 1) no multidetector HRCT data available (n = 133), 2) HRCT study performed more than 3 months before or after the data of the surgical lung biopsy (n = 20) and 3) histopathologic diagnosis assigned was not UIP, fibrotic NSIP or CHP (n = 23). The final study population was made up of 162 patients. Seventeen patients had a biopsy taken at two different sites. The MDT diagnoses were as follows: idiopathic NSIP (n = 25), IPF/UIP (n = 66), connective tissue disease related NSIP (n = 18), connective tissue disease related UIP (n = 4) and CHP (n = 49). Complete pulmonary function tests within 3 months of the biopsy date were available in 117 patients. Of the 162 patients enrolled in the study, 60 were female. Mean age at the time of surgical lung biopsy was 61.3 years (SD = 13.4). The median interval between surgical lung biopsy and HRCT was 45 days (IQR = 21.4). A summary of the patient demographics including mean HRCT scores and median histopathologic scores is shown in Table 1. Interobserver agreement for FF scores and traction bronchiectasis was good (wκ = 0.69 and wκ = 0.63, respectively). Interobserver agreement for HRCT pattern scores measured using the single determination standard deviation ranged from 1.2 % (total disease extent score) to 6.9 % (extent of reticulation).

**Table 1:** Patient demographics, mean ± SD HRCT scores and median (range) histopathologic scores in MDT diagnosis subgroups

	*Fibrotic NSIP	*IPF/UIP	CHP
	(n=43)	(n=70)	(n=49)
Age	52.3±13.2	62.3±10.2	65.3±14.2
Gender (male/female)	19/24	58/11	25/24
Smoking (never/ever/current)	25/16/2	24/6/4	35/13/1
Total disease extent	31.5±22.5	36.3±20.4	37.6±28.3
Groundglass opacification	17.2±15.4	14.5±15.8	25.5±27.5
Reticulation	13.1±12.7	18.5±10.9	9.6±9.7
Honeycombing	0.45±1.9	2.2±5.9	1.2±5.4
Consolidation	0.1±0.1	0.1±0.1	0.2±0.1
Emphysema	0.49±1.76	1.2±4.1	0.9±4.3
Traction bronchiectasis	1.0±0.9	1.7±1.8	1.1±1.7
FF score	0.0 (0–1)	2.5(0–5.5)	1.5(0–5)

*NSIP =idiopathic non-specific interstitial pneumonia and connective tissue disease related non-specific interstitial pneumonia combined, *IPF/UIP =idiopathic pulmonary fibrosis and connective tissue disease related usual interstitial pneumonia combined, CHP=chronic hypersensitivity pneumonitis

### Relationships between fibroblastic foci score and HRCT patterns in fibrotic lung disease

On univariate analysis, the strongest correlation was between FF scores and traction bronchiectasis scores. Weaker, but significant correlations were also demonstrated between FF score and extent of reticulation, and FF score and extent of honeycombing (Table 2). Multivariate regression analysis was performed using FF score as the dependent variable and total disease extent on HRCT, the four interstitial HRCT patterns (extent groundglass opacification, reticulation, honeycombing, consolidation) and traction bronchiectasis scores as the independent variables. Only increasing extent of reticulation and increasing traction bronchiectasis scores were independently correlated with FF score (traction bronchiectasis: *P* < 0.0001, 95%CI 0.27–0.53, reticulation *P* < 0.0001, 95%CI 0.01–0.04). On re-analysis, omitting 4 outliers identified by inspecting leverage-versus-residual squared plots, the same variables were retained in the equation with the same statistical significance (traction bronchiectasis, *P* < 0.0001, extent of reticulation, *P* < 0.0001).

**Table 2:** Univariate correlations between FF scores and HRCT variables expressed as Spearman’s rank correlation coefficient (n=162)

	FF score	
	r	*P* value
Total disease extent	0.10	0.22
Groundglass opacification	−0.09	0.21
Reticulation	0.42	<0.0001
Honeycombing	0.21	0.01
Consolidation	0.04	0.58
Traction bronchiectasis	0.50	<0.0001

To determine whether this link was enhanced by localizing the HRCT pattern scores to the biopsy site, the following analysis was performed: 24 cases had biopsies taken from either the right or left upper lobe and 40 had biopsies taken from the right lower or left lower lobe. Total disease extent scores, all HRCT pattern scores and traction bronchiectasis scores were recalculated for the upper two levels (‘upper zone’ scores) and for the lower two levels (‘lower zone’ scores). The analysis was repeated within these subgroups with the appropriate ‘upper zone’ or ‘lower zone’ HRCT variables. In both analyses, the explanatory power of traction bronchiectasis increased when compared to the analysis performed using ‘whole lung’ HRCT variables and was also the only independent predictor of FF score (upper zone: r^2^ = 0.56, *P* < 0.0001, 95%CI 0.32–0.86, lower zone: r^2^ = 0.48, *P* < 0.0001, 95%CI 0.34–0.74).

### Relationships between fibroblastic foci score and HRCT patterns in fibrotic lung disease subgroups

The same analysis was performed in each diagnostic subgroup. On univariate analysis, significant correlations were identified between FF score and traction bronchiectasis scores in patients with IPF/UIP, CTD related UIP, and CHP (Table 3). Increasing extent of reticulation also correlated with increasing FF score in patients with CHP. On multivariate regression analysis the only significant independent predictors of increasing FF score were increasing severity of traction bronchiectasis in patients with IPF/UIP (n = 66, r^2^ = 0.19, *P* < 0.0001) and patients with CHP (n = 49, r^2^ = 0.45, *P* < 0.0001). To determine if patients with a histopathologic pattern of UIP within the CHP group were disproportionately impacting this observation, the CHP group was separated into patients assigned a diagnosis of CHP on histopathologic analysis (n = 29) and those with a histopathologic pattern other than CHP (UIP-like = 16, NSIP-like = 4). In this analysis severity of traction bronchiectasis was the only HRCT pattern which independently correlated with FF score in patients with concordant histopathologic and MDT diagnoses of CHP (r^2^ = 0.64, *P* < 0.0001).

**Table 3:** Univariate correlations between FF scores and HRCT variables expressed as Spearman’s rank correlation coefficient, in patient MDT diagnosis subgroups

	*Fibrotic NSIP	IPF/UIP	CHP
	(n=43)	(n=70)	(n=49)
Total disease extent	0.04 (0.80)	0.12 (0.31)	0.02 (0.88)
Groundglass opacification	−0.07 (0.64)	0.10 (0.42)	−0.15 (0.31)
Reticulation	0.15 (0.34)	0.22 (0.07)	0.52 (0.001)
Honeycombing	0.18 (0.05)	−0.06 (0.62)	0.372 (0.01)
Consolidation	0.02 (0.75)	0.09 (0.45)	−0.02 (0.85)
Traction bronchiectasis	0.60 (0.77)	0.44 (<0.0001)	0.61 (<0.0001)

Numbers in parentheses are *P* values. *NSIP =idiopathic non-specific interstitial pneumonia and connective tissue disease related non-specific interstitial pneumonia combined, *IPF/UIP =idiopathic pulmonary fibrosis and connective tissue disease related usual interstitial pneumonia combined, CHP=chronic hypersensitivity pneumonitis

### Variables correlated with severity of traction bronchiectasis patterns in disease subgroups

Variables which independently correlated with severity of traction bronchiectasis were determined by the same methods above, using severity of traction bronchiectasis as the dependent variable. Three separate subgroups were evaluated:IPF/UIP (n = 70), a combined group of idiopathic fibrotic NSIP and connective tissue disease related NSIP (n = 43) and CHP (n = 49). In patients with IPF and CHP, only FF score correlated with severity of traction bronchiectasis. In the combined fibrotic NSIP group, only increasing total disease extent and decreasing extent of emphysema independently correlated with severity of traction bronchiectasis (Table 4). When the CPI was included in each of these analyses (representing global physiologic disease severity), the same variables were retained in each equation with the same statistical significance.

**Table 4:** Correlation of severity of traction bronchiectasis with HRCT pattern extents and fibroblastic foci scores on multivariate linear regression within disease subgroups

	HRCT pattern	Partial regression coefficient	*P* value	95 % CI
*IPF/UIP (n=70) r^2^ = 0.34	FF score	0.49	<0.0001	0.17–0.80
	Global disease extent	−0.02	0.74	−0.12–0.08
	Extent of reticulation	0.04	0.03	0.00–0.08
	Extent of ground glass	0.01	0.09	−0.02–0.03
	Extent of honeycombing	0.02	0.61	−0.05–0.08
	Extent of consolidation	−0.26	0.63	−1.30–0.79
	Extent of emphysema	−0.31	0.05	−0.60– −0.01
*Fibrotic NSIP (n=43) r^2^ = 0.58	FF score	0.29	0.29	−0.27–0.86
	Global disease extent	0.03	<0.0001	0.02–0.69
	Extent of reticulation	0.03	0.27	−0.02–0.08
	Extent of ground glass	0.004	0.60	−0.01–0.02
	Extent of honeycombing	0.01	0.76	−0.07–0.09
	Extent of consolidation	0.04	0.74	−0.66–0.91
	Extent of emphysema	−0.31	0.04	−0.60– −0.01
CHP (n=49) r^2^ = 0.35	FF score	0.72	<0.0001	0.43–1.00
	Global disease extent	−0.02	0.67	−0.13–0.08
	Extent of reticulation	0.03	0.27	−0.02–0.08
	Extent of ground glass	0.004	0.60	−0.01–0.02
	Extent of honeycombing	0.01	0.76	−0.07–0.09
	Extent of consolidation	0.04	0.74	−0.66–0.91
	Extent of emphysema	−0.73	0.05	−1.46– −0.01

*Fibrotic NSIP group = idiopathic fibrotic NSIP (n=25) and CTD-NSIP (n=18) combined. NSIP= non-specific interstitial pneumonia. *IPF/UIP =idiopathic pulmonary fibrosis and connective tissue disease related usual interstitial pneumonia combined. CHP=chronic hypersensitivity pneumonitis

## Discussion

Our study has demonstrated, for the first time, that fibroblastic foci profusion on histopathologic analysis correlates most strongly with severity of traction bronchiectasis shown on HRCT images. On analysis of subgroups stratified according to MDT diagnosis, severity of traction bronchiectasis remained the only HRCT pattern that correlated with fibroblastic foci profusion in patients with an MDT diagnosis of IPF and CHP.

The histopathologic appearances of UIP are characterized by a temporally and spatially heterogeneous intermix of normal lung, well-established acellular bundles of collagen with microscopic honeycombing and areas of new myxoid matrix containing aggregates of actively proliferating and collagen-producing myofibroblasts, the so-called ‘fibroblastic foci’. These foci are clinically important because in IPF their profusion on surgical lung biopsy predicts physiologic decline and mortality [7, 8, 26, 27]. Over the last 10 years, there has been a growing reliance on clinical and chest-imaging criteria to diagnose IPF, with surgical lung biopsy usually being reserved for cases in which the diagnosis remains unclear on clinical and imaging grounds alone. This shift in diagnostic thinking has primarily been driven by changes in perception regarding histopathology as a diagnostic gold standard, as well as the widely accepted observation that in idiopathic fibrotic lung disease a typical HRCT pattern of UIP is sufficient to secure a diagnosis of IPF in the majority of cases [23, 28, 29]. Consequently, the clinical applicability of fibroblastic foci profusion as a marker of prognosis is limited to a small minority of patients. By contrast, HRCT imaging is routinely performed in patients with diffuse lung disease, and several HRCT patterns, in particular honeycombing and, more recently, traction bronchiectasis, have been reported as prognostically important in several different fibrotic lung diseases [2, 4–6, 21, 22]. Based upon these observations, we formed an a priori hypothesis that one or more individual HRCT patterns may be a surrogate marker of fibroblastic foci profusion.

The key observation in our study was that severity of traction bronchiectasis correlates with fibroblastic foci profusion on histopathologic analysis, which confirms our original hypothesis. This finding was reinforced by three separate observations. First, when a subgroup analysis of patients who had upper or lower lobe surgical lung biopsies was performed using modified ‘upper zone’ and ‘lower zone’ traction bronchiectasis scores respectively, the explanatory power of traction bronchiectasis scores was improved when compared to traction bronchiectasis scores generated from all six levels of the lungs. Second, on two multivariate analyses, adjusted for disease severity, first using total disease extent on HRCT and second using the CPI, traction bronchiectasis was the only variable which independently correlated with fibroblastic foci profusion. In this analysis, the relationship between fibroblastic foci profusion and severity of traction bronchiectasis was not influenced by the mode of scoring global disease severity. Third, when predictors of severity of traction bronchiectasis were investigated using traction bronchiectasis as the dependent variable, fibroblastic foci score was the variable which most strongly correlated.

Once the FF-traction bronchiectasis relationship had been established within the entire cohort, it was predictable that it remained robust in patients with an MDT diagnosis of IPF. Both fibroblastic foci profusion and traction bronchiectasis have been shown to be important predictors of mortality in patients with IPF, and IPF patients within our cohort had the highest mean traction bronchiectasis scores and highest median FF scores, thus empowering this subgroup for analysis [2, 21, 22].

The relationship between severity of traction bronchiectasis and fibroblastic foci profusion in patients with an MDT diagnosis of CHP was less expected. It is well recognized that patients with an MDT diagnosis of CHP may have pathologic appearances which are identical to those of UIP [15, 30, 31]. Based upon the established relationship between traction bronchiectasis and FF scores in patients with IPF, we performed a subgroup analysis within the CHP group, stratifying by pathologic appearance, to determine if an underlying pattern of UIP was disproportionately impacting our observations in this CHP group. Although the FF-traction bronchiectasis relationship remained robust in patients whose biopsies had specifically been assigned an MDT diagnosis of CHP, even in these cases, the distinction between UIP and CHP might often be considered a matter of fine judgment — one that is determined only by variable degrees of inflammation, granuloma formation and bronchocentricity (depending on how active the HP component is). Relatively few studies have evaluated the frequency of CHP on histopathologic analysis in the setting of an MDT diagnosis of CHP, and those that have are limited by small patient numbers [15, 31]. In a study involving 25 cases of CHP diagnosed on clinico-radiologic-pathologic grounds, Churg et al. reported that 18 (72 %) had a pathologic pattern of fibrosis predominantly suggestive of UIP, with some added bronchocentric fibrosis associated with fibroblastic foci [15]. The remaining 7 cases had NSIP-like appearances (n = 4) or showed only bronchocentric fibrosis (n = 3). Our results in the CHP subgroup may reflect the degree of overlap that exists between the pathologic appearances of UIP/IPF and CHP. It is noteworthy that although there were only 4 cases of connective tissue related UIP, at least on univariate analysis, a significant correlation was demonstrated between FF score and severity of traction bronchiectasis in these patients. It is possible that the relationship between FF score and severity of traction bronchiectasis we have observed is confined to patients with UIP.

Our findings in the fibrotic NSIP subgroup support this conclusion. A key feature that distinguishes fibrotic NSIP from UIP is the relative paucity of fibroblastic foci seen in the former. The purpose of including patients with a histopathologic diagnosis of fibrotic NSIP in our analysis was to ensure that the full range of possible FF scores (including a score of zero) was captured. In the current study, of the 25 patients with an MDT diagnosis of idiopathic NSIP, 20 cases were assigned an FF score of zero by at least one pathologist (12 by both pathologists). Of the 18 patients in the connective tissue disease related fibrotic NSIP group, 15 cases were assigned an FFscore of zero by at least one pathologist (10 by both pathologists). Consequently, in both idiopathic and connective tissue disease related fibrotic NSIP, no relationship between severity of traction bronchiectasis and fibroblastic foci profusion was identified on any of the analyses. In a recent study of patients with connective tissue disease related fibrotic lung disease, moderately high traction bronchiectasis scores (using the same method of scoring as the present study) were reported in a subgroup (n = 26) of patients with biopsy proven fibrotic NSIP [4]. In the current study, when combining patients with idiopathic and connective tissue related fibrotic NSIP, disease severity on HRCT, reflected by total disease extent scores, correlated with the severity of traction bronchiectasis rather than fibroblastic foci profusion. Thesedata, as well as anecdotal evidence, suggest that there are other factors, possibly global disease severity, which play a more significant role than fibroblastic foci profusion alone in determining severity of traction bronchiectasis in patients with fibrotic NSIP.

It is important to highlight that the results of this study do not indicate a causal relationship between fibroblastic foci profusion and severity of traction bronchiectasis. This is supported, first by the observation that no significant correlation between FF score and severity of traction bronchiectasis in patients with fibrotic NSIP was demonstrated, and second by well-established observations of severe traction bronchiectasis in disease entities in which fibroblastic foci are not generally considered a major component, such as end stage fibrotic sarcoidosis, drug-induced fibrosis or radiation-induced fibrosis. While the contractile forces of fibroblastic foci may contribute to severity of traction bronchiectasis as our results suggest, the relationship is clearly not straightforward.

Our study has some limitations. It was retrospective in design, and spanned 13 years, over which time there have been changes in diagnostic guidelines regarding fibrotic lung disease subsets, especially regarding the role of CT in the diagnosis of IPF. However, all diagnoses were made based on currently accepted histopathologic criteria and following MDT collaboration, which is now regarded as the diagnostic gold standard for diffuse lung diseases [23]. Another possible limitation of the study is that all of the patients underwent surgical lung biopsy, meaning that imaging and clinical parameters were insufficient for a secure diagnosis to be made. Consequently, our study cohort may not replicate an unselected patient population with fibrotic lung disease, in which only a small proportion of patients usually proceed to biopsy for diagnosis. We emphasize, however, that this was an HRCT-histopathologic correlative study with a specific aim: to identify links between fibroblastic foci and HRCT patterns in fibrotic lung disease. Only 17 of the 162 patients had surgical lung biopsies taken at more than one site and fibroblastic foci were scored using a semiquantitative method. This limited sampling and method of scoring could have impactedFF score accuracy. It is worth highlighting that quantification of fibroblastic foci, which most likely represent a three-dimensional structure within the lung on two-dimensional biopsy slides, may also impact accuracy [32]. Regardless, all of the biopsy specimens were evaluated by pathologists with expertise in the histopathology of interstitial lung disease.

## Conclusions

In conclusion, we have demonstrated that the severity of traction bronchiectasis shown on HRCT independently predicts fibroblastic foci profusion in patients with IPF and CHP. This finding possibly explains the growing evidence that severity of traction bronchiectasis is an important predictor of prognosis in several different fibrotic lung diseases.

## Abbreviations

CHP: chronic hypersensitivity pneumonitis
CPI: composite physiological index
CTD: connective tissue disease
FF: fibroblastic foci (score)
HRCT: high resolution computed tomography
IPF: idiopathic pulmonary fibrosis
NSIP: non-specific interstitial pneumonia
UIP: usual interstitial pneumonia
